# The Effects of the Light Spectral Composition on the Development of Olive Tree Varieties Mediated by Photoreceptors

**DOI:** 10.3390/ijms26178319

**Published:** 2025-08-27

**Authors:** Ivano Forgione, Ida Quattromano, Teresa Maria Rosaria Regina, Amelia Salimonti, Fabrizio Carbone

**Affiliations:** 1 Research Center for Olive, Fruit and Citrus Crops, Council for Agricultural Research and Economics (CREA), Via Settimio Severo 83, 87036 Rende, CS, Italy; 2 Department of Biology, Ecology and Earth Science, University of Calabria—Rende, 87036 Rende, CS, Italy

**Keywords:** *Olea europaea*, phenotyping, red and blue light, photoreceptors, gene expression

## Abstract

Plants have the ability to perceive a wide range of light spectra, from which they derive not only the energy required for photosynthesis but also a variety of environmental cues and signals mediated by specific photoreceptors that trigger a cascade of biochemical reactions essential for their development. The olive tree (*Olea europaea* L.) is a woody species for which, despite its agronomic and economic relevance, the influence of light on its development remains poorly understood. The present study, a combined approach was employed, involving the phenotyping of 10 different cultivars exposed exclusively to red light (RL) and blue light (BL) for a period of two months, in addition to the monitoring of expression profiles of 10 photoreceptor-encoding genes in two of the cultivars that exhibited the most contrasting responses to the different light conditions. Our results revealed a correlation between the expression of specific genes and the differential response to exclusive exposure to the two light spectra, highlighting a generally enhanced photosynthetic activity of nearly all cultivars to blue light (BL) and, conversely, a negative response to red light (RL). Taken together, our data, by elucidating the response of the olive to specific light spectra and the underlying molecular mechanisms, pave the way for further studies on these traits, which could be useful for the improvement of this species.

## 1. Introduction

Olive (*Olea europaea* L.) is a tree crop widely spread in the countries of the Mediterranean basin and it is cultivated for table olive and oil production.

The growth of olive plants from seeds is very slow and characterized by an extended juvenile phase [1] lasting 10 to 15 years, consisting solely of vegetative proliferation and the absence of flowers and fruits. This characteristic of the species may impose economic constraints on farmers. The irregular fruiting conferred by the alternate bearing depends on physiological, environmental and genetic factors [2] and has a significant economic impact on olive cultivation as well. Olive propagation is based mostly on self-rooted cuttings and by using rootstock that can be originated either from seeds grown plants or specific cultivars. In the case of olives that have been traditionally trained, the shape is determined by pruning activity. The purpose of this activity is twofold: firstly, to facilitate the harvesting of fruit and, secondly, to produce a canopy that is predominantly characterized by an open centre or vase in order to increase exposure to light.

Although intensively cultivated olives require planting distances to ensure good exposure to sunlight and avoid reciprocal shading of adjacent trees, in the last two decades, modern orchards with high plant density have been widely used to get stable yield and facilitate harvesting [3].

Light is indeed one of the most important environmental factors that plants use to produce chemical energy, which through photosynthesis reactions is converted into sugars utilized as nutrition for growth and development [4]. In addition to the different photosynthetic pigments that absorb light at different wavelength, plants are provided with special receptors that in response to their photoactivation can modulate a wide number of processes at both intracellular and extracellular level. Plant photoreceptors are chromoproteins that transition to their active state upon the perception of light by the chromophore. Subsequent to this, biochemical signals are received and gene expression is modulated, thereby triggering physiological responses. Phytochromes (PHYs) are the wider class of photoreceptors in the red and far-red wavelength [5], cryptochromes (CRYs) respond to UV-A and blue light [6] as well as phototropins (PHOTs) [7], whereas UVR8 mediates the perception of both UV-B and UV-A [8]. The photoreceptors allow plants to discriminate different light conditions and detect quantity, quality and duration.

Many studies have demonstrated that different light quality have effects on several physiological events, such as phototropism, chloroplast movements and orientation, stomatal opening and photosynthetic activity [9] on model organisms [10] and horticultural plants [11,12,13,14]. However, knowledge regarding woody species remains limited. Fernbach and Mohr (1990) [15] elucidated the role of the phytochrome in the absorption of far-red light in *Pinus sylvestris* L. hypocotyl growth. Experiments conducted in a Japanese pear orchard revealed that the addition of far-red light caused early flower initiation by terminal buds, reduced the accumulation of cytokinin and gibberellic acid [16]. The study of photosynthetic parameters is a valuable approach to understanding the physiological effects on plants of different light spectra. On three woody species *Populus* × *canadensis*, *Quercus ilex* and *Citrus reticulata* blue light at high intensity reduced photosynthesis, stomatal conductance and mesophyll conductance [17].

As is the case with the majority of woody crops, the impact of light on olive growth remains to be fully elucidated. However, a recent report indicated that fruits harvested from light-irradiated branches of ‘Leccino’ exhibited superior performance in terms of fresh weight, dry weight, fruit size, mesocarp thickness and cell size when compared to those harvested from shaded branches [18]. In vitro experiments revealed that light quality also affected the olive seedlings growth which exhibited increased stem length and internodal elongation when irradiated with high red/blue ratio LED [19]. Our recent study highlighted the different vegetative activity under extended photoperiod in olive and interesting rhythmic expression of specific genes. We found that olive plants exposed to continuous light exhibited increased growth than light/darkness regime and the fastest growth was detected early in the first thirty days of treatment. Additionally, many diurnal oscillating genes related to circadian clock, fatty acids and phenylpropanoids maintained rhythmicity after one and two months of continuous light exposure [20]. Our additional study revealed that light works as regulator of mediators such as photoreceptors, in a complex network that modulates the production of photoprotective pigments and lipid biosynthesis [21].

In order to investigate the effects of different light qualities on olive plants, under controlled environmental conditions in a photoperiod of 16/8 h light/darkness we irradiated ten different cultivars with exclusively red (RL) and blue (BL) light. To simulate the natural conditions, the plants were exposed to white light (WL) as control. All plants were subjected to a detailed analysis of photosynthetic activity through a photosynthesis portable system and to constant monitoring of their growth to evaluate their performance under the different treatments. RT-PCR analyses of transcripts encoding specific photoreceptors provided useful information about their functional activation and putative crosstalk in response to different light quality.

## 2. Results

### 2.1. Physiological Phenotyping of Light Quality Response on Olive Cultivars

To highlight the differential responses under different light spectra of ten (10) cultivars (Arbequina, Canino, Coratina, Frantoio, FS17, Itrana, Leccino, Maurino, Moraiolo and Pendolino), we exposed six-months-old plants RL andBL and we monitored both biometric and physiological parameters for thirty (T1) and sixty days (T2) after the start of treatment (T0) (Figure S1).

#### 2.1.1. Growth Responses Mediated by Blue and Red Lights

**Figure 1:**
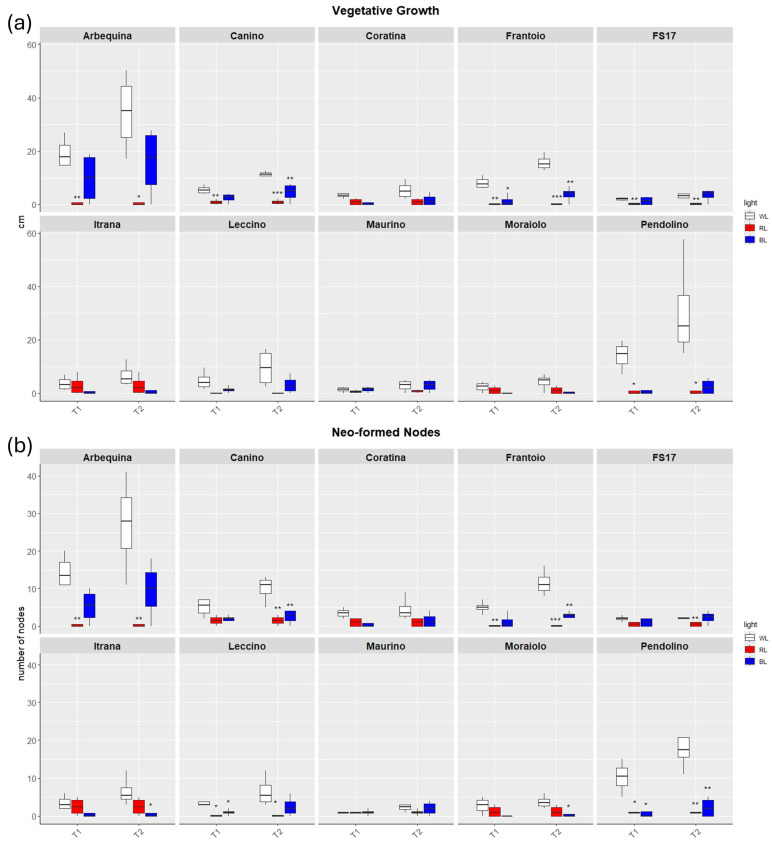
Boxplots of vegetative growth (**a**) and neo-formed nodes (**b**) in the ten cultivars measured at T1 and T2. White, red and blue boxplots indicate WL, RL and BL treatment, respectively. Black asterisks on red and blue boxplot indicate significant differences in comparison to WL of the same timepoint. Statistical analysis is performed according to Student’s *t*-test (* *p* ≤ 0.05, ** *p* ≤ 0.01, *** *p* ≤ 0.001).

The length of new sprouts and the number of neo-formed nodes were measured at T1 and T2. At T1, under control white light (WL), the measurements of the shoot leader elongation and the neo-formed lateral shoots evidenced the best performance of the cv Arbequina with a mean growth of 19.2 cm after thirty days from T0, followed by ‘Pendolino’ and ‘Frantoio’ with a growth of 14 and 8 cm, respectively (Figure 1).

Analysis of the vegetative growth and neo-formed nodes suggested that plants under BL grew better than those under RL demonstrating a greater similarity to the behavior of control plants.

**Figure 2:**
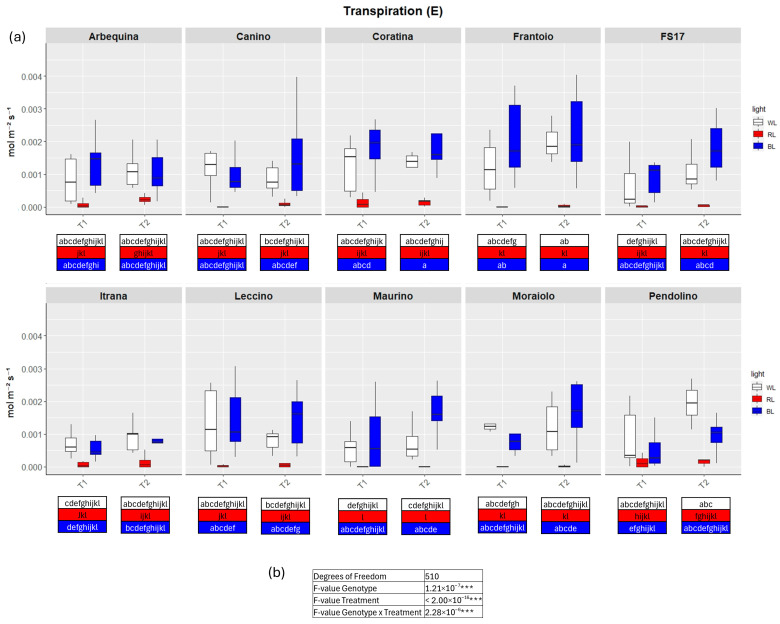
(**a**) Boxplots of transpiration—E in the ten cultivars measured at T1 and T2. White, red and blue boxplots indicate WL, RL and BL treatment, respectively. Different letters below each sample in the two time points indicate significant differences among all samples according to Tukey’s test (*p* < 0.05). (**b**) Two-way ANOVA is performed considering genotype, treatment and genotype × treatment, *** *p* ≤ 0.001).

Thirty-days (T1) exposure to BL negatively affected the appearance of new sprouts and limited the elongation of the shoot leader than WL only in ‘Frantoio’ and ‘Pendolino’ (Figure 1), as well as a decreasing trend in the production of neo-formed nodes was detected with significant differences for ‘Leccino’ and ‘Pendolino’ (Figure 2).

RL significantly reduced the formation and elongation of sprouts than WL in ‘Arbequina’, ‘Canino’, ‘Frantoio’, ‘FS17’, and ‘Pendolino’. The only cultivar which showed different behavior respect to the other cultivars was ‘Itrana’ with not significant growth differences between RL and WL (Figure 1). A similar trend was observed in the neo-formed nodes with a significantly lower number of them in ‘Arbequina’, ‘Frantoio’, ‘Leccino’ and ‘Pendolino’ treated with RL in contrast to WL (Figure 2).

After the exposure to additional 30 days (T2), plants in BL evidenced vegetative activity with an increasing trend from T1 to T2 in all the cultivars, even though not statistically significant. Conversely, the majority exhibited a reduced growth rate respect to the control plants, although the difference in the Pendolino cultivar was more pronounced and evident (Figure 1). Similarly to vegetative growth, also neo-node formation in BL tended to increase from T1 to T2 in all the cultivars with significant lower number of neo-formed nodes detected in ‘Canino’, ‘Frantoio’, ‘Itrana’, ‘Moraiolo’ and ‘Pendolino’.

The exposure to RL until T2 maintained the growth and proliferation blocked like at T1. Here we didn’t observe any vegetative activity in all the cultivars analyzed.

#### 2.1.2. Quantification of Photosynthetic Efficiency Affected by Light Quality

The same cultivars were also monitored in order to investigate the effects that different light spectra might have on some aspects of photosynthesis. Transpiration rate (E), CO_2_ assimilation rate (A), stomatal conductance (GSW) and electron transport rate (ETR) were measured by a portable gas exchange system (LiCor LI-6800) on plants grown under BL and RL.

**Figure 3:**
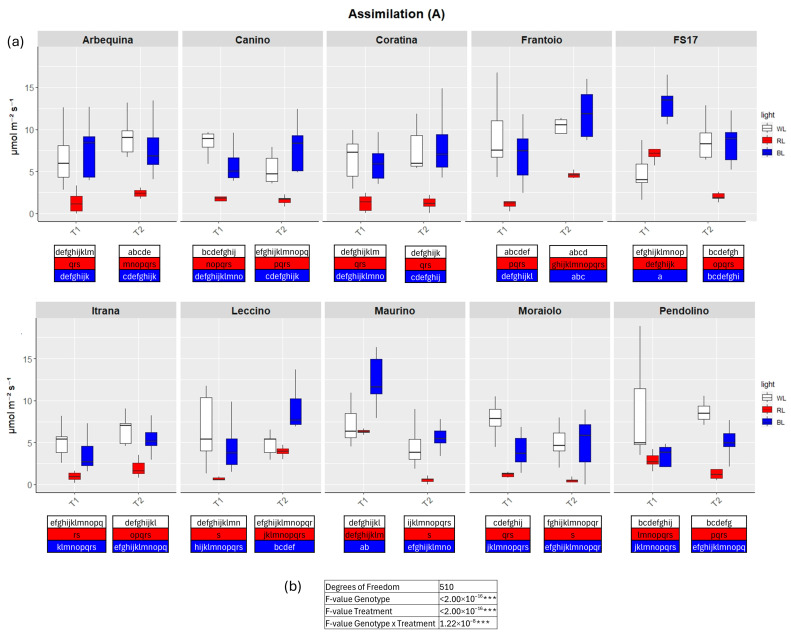
(**a**) Boxplots of CO_2_ assimilation rate—A in the ten cultivars measured at T1 and T2. White, red and blue boxplots indicate WL, RL and BL treatment, respectively. Different letters below each sample in the two time points indicate significant differences among all samples according to Tukey’s test (*p* < 0.05). (**b**) Two-way ANOVA is performed considering genotype, treatment and genotype × treatment (*** *p* ≤ 0.001).

**Figure 4:**
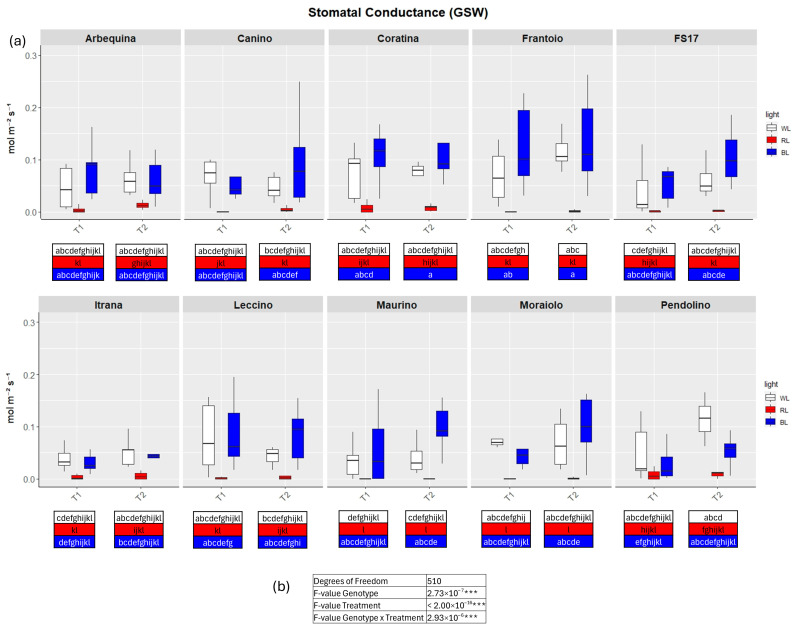
(**a**) Boxplots of stomatal conductance—GSW in the ten cultivars measured at T1 and T2. White, red and blue boxplots indicate WL, RL and BL treatment, respectively. Different letters below each sample in the two time points indicate significant differences among all samples according to Tukey’s test (*p* < 0.05). (**b**) Two-way ANOVA is performed considering genotype, treatment and genotype × treatment (*** *p* ≤ 0.001).

**Figure 5:**
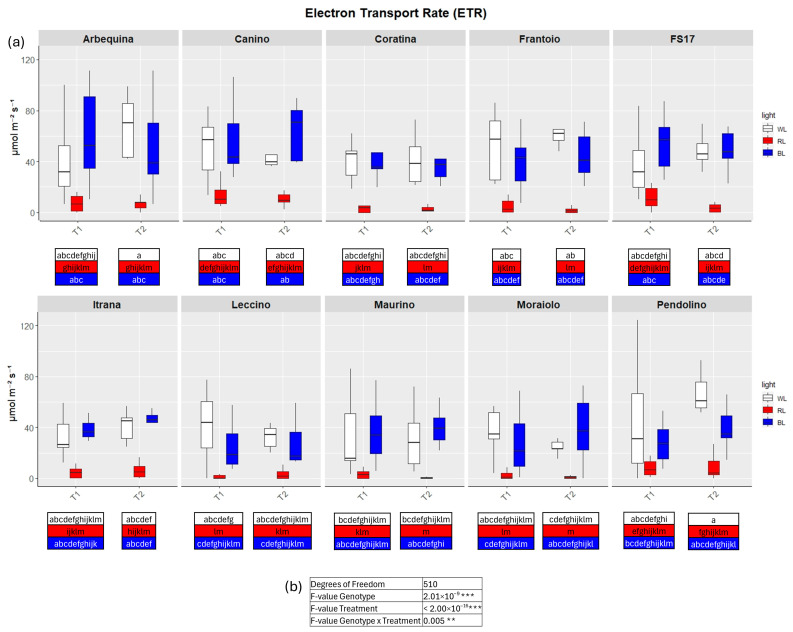
(**a**) Boxplots of electron transport rate—ETR in the ten cultivars measured at T1 and T2. White, red and blue boxplots indicate WL, RL and BL treatment, respectively. Different letters below each sample in the two time points indicate significant differences among all samples according to Tukey’s test (*p* < 0.05). (**b**) Two-way ANOVA is performed considering genotype, treatment and genotype × treatment (** *p* ≤ 0.01, *** *p* ≤ 0.001).

TWO-way analysis of variance (ANOVA) evidenced significant differences for all four parameters when the variable Genotype and Treatment were separately considered as well as significant differences were detected when interaction between Genotype and Treatment were explored by the two-way ANOVA (Figure 2, Figure 3, Figure 4 and Figure 5b).

In detail, in BL all measured parameters followed the WL trend, like biometric observations (Figure 2, Figure 3, Figure 4 and Figure 5a, Figures S2–S5). At T1 the only significant differences concerned A, which was higher in ‘FS17’ and ‘Maurino’ (Figure 3a and Figure S3a).

There were no significant differences between BL and WL at T2 in all parameters analyzed (Figure 2, Figure 3, Figure 4 and Figure 5a, Figures S2–S5).

In contrast to BL, RL exerted a negative influence on photosynthetic efficiency, evidenced by a substantial decline in the parameters E and GSW in ‘Frantoio’ and ‘Moraiolo’ at T1 (see Figure 2 and Figure 4a, Figures S1a and Figure S3a). Furthermore, ETR was found to be diminished in ‘Canino’, ‘Frantoio’, and ‘Leccino’ at T1 (see Figure 5a and Figure S4a); while A exhibited a reduction in all cultivars, excluding ‘FS17’ and ‘Maurino’ (Figure 3a and Figure S3a).

At T2 RL reduced E and GSW in ‘Frantoio’ and ‘Pendolino’ (Figure 2 and Figure 4a, Figure S2b and Figure S4b); ETR in ‘Arbequina’, ‘Canino’, ‘Frantoio’, ‘FS17’ and ‘Itrana’ (Figure 5a and Figure S5b); A in all the cultivars except ‘Canino’, ‘Leccino’ and ‘Maurino’ (Figure 3a and Figure S3b).

To get a wider overview about the whole-time span of the experiment we compared values of each parameter between T1 and T2. The comparison between measurements for A in BL of T1 and T2 evidenced an ascending trend in ‘Frantoio’ and ‘Leccino’, and a descending trend in ‘Maurino’ and ‘FS17’ (Figure 3a and Figure S3). A significant descending trend was also observed from T1 to T2 in the latter two cases (Figure 3a and Figure S3).

**Figure 6:**
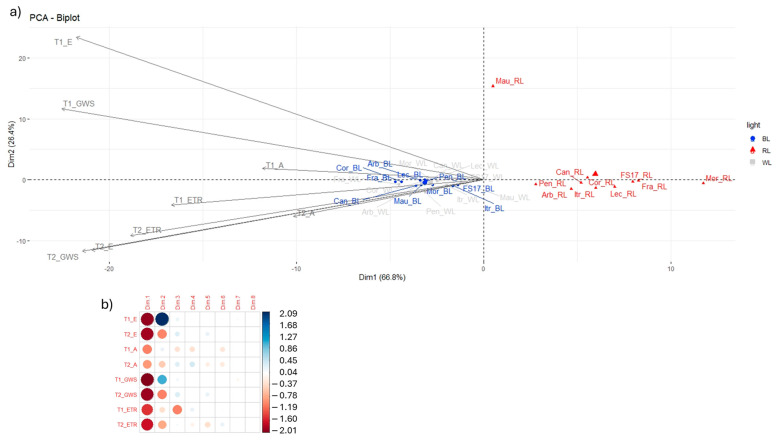
(**a**) Principal component analysis of LiCor parameters with depiction of first two principal components. (**b**) Correlation between LiCor parameters and components, correlation values range from −2 (strong negative correlation) to +2 (strong positive correlation).

As previously outlined, the effects of differing light quality treatments on olive photosynthetic activity were found to be significant. A principal component analysis (PCA) was performed on the four parameters (E, A, GSW and ETR) collected by the LiCor 6800 in order to ascertain the influence of light treatments on olive plant physiology. This analysis enabled the identification of the more negative effect of RL exposure on photosynthesis efficiency along the first component (Dim1) (Figure 6b). In fact, PCA showed a clear separation of the RL treated samples, with the peculiar behavior of ‘Maurino’ which clearly segregated by RL cluster on the y axis (Dim2) (Figure 6b). PCA also evidenced the effect BL observed for growth and photosynthesis, indeed, WL and BL samples overlapped along the Dim1, accounting for 66.8% of the total variation. This was highly correlated with E, GSW and ETR at both T1 and T2, with a weaker contribution from A (Figure 6a). Dim2 explained 26.4% of the variation and was attributed largely to E at T1, and slightly less to E at T2, GSW at both T1 and T2 and ETR at T2 (Figure 6a).

### 2.2. Gene Expression Analysis of Photoreceptors in Contrasting Light-Responsive Cultivars

In contrast to the repression of growth and photosynthesis caused by RL, BL has been observed to stimulate olive plants by activating specific responses to particular light wavelengths. These responses are likely modulated by genes that encode photoreceptors for blue light. This prompted an evaluation of the expression of blue-light receptor genes and red-light signaling mediators that were putatively involved in such regulation in two contrasting cultivars, in relation to their response to blue light. ‘Canino’ was selected for its good blue light response according to biometric and physiological data and ‘Pendolino’ as counterpart.

For this reason, leaves were sampled from plants of both genotypes, grown for two months (T2) in different experimental conditions (WL, BL and RL). The transcript levels of ten genes directly involved in photoperception processes were monitored: among blue-light receptors genes we selected two genes coding for *phototropin* 1 and *phototropin* 2 respectively (*PHOT1* and *PHOT2*), two genes coding for *cryptochrome* 1 and *cryptochrome* 2 (*CRY1* and *CRY2*); among mediator genes of the red-light signaling we analyzed four genes coding for *phytochromes A*, *B*, *C* and *E* (*PHYA, PHYB, PHYC, PHYE*); moreover, further two genes that encode two photoreceptors or presumed such containing the *Photolyase Homology Region* domain common to *photolyases* and *cryptochromes* (*PHR* and *PHR2*), were added to our analyses.

**Figure 7:**
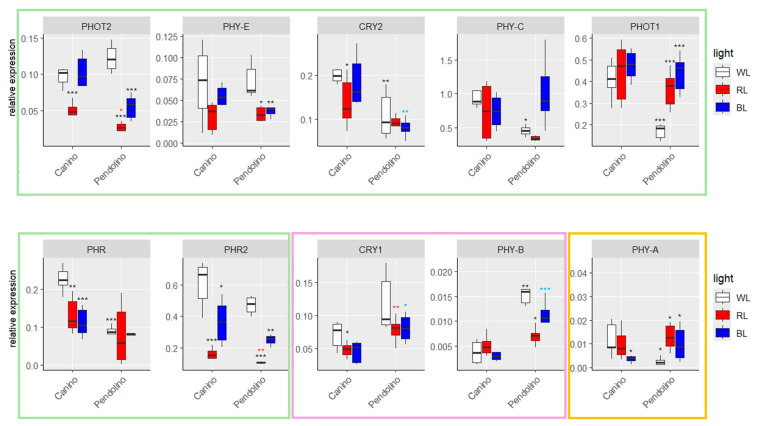
Boxplots of RT-qPCR in ‘Canino’ and ‘Pendolino’ at T2. White, red and blue boxplots indicate WL, RL and BL treatment, respectively. Black asterisks on red and blue boxplot indicate significant differences in comparison to WL of the same cultivar. Black asterisks on WL, red asterisks on RL and blue asterisks on BL indicate significant difference between the two cultivars at the same treatment. Statistical analysis is performed according to Student’s *t*-test (* *p* ≤ 0.05, ** *p* ≤ 0.01, *** *p* ≤ 0.001). Green, pink and yellow boxes grouped genes with similar expression trends among cultivars.

Seven out of ten monitored genes, *PHOT2, PHYE, CRY2, PHYC, PHOT1, PHR and PHR2*, shared a similar trend potentially associated with the increased capacity in BL responding of blue light-sensitive ‘Canino’ than of ‘Pendolino’ (Figure 7, Table S1).

In detail, it was observed that the expression of *PHOT2* and *PHYE* in ‘Canino’ plants exposed to BL remained constant compared to the control plants (WL); on the contrary, its expression was significantly reduced in plants exposed to RL. Conversely, a significant decrease in the transcript levels of these genes was observed in plants exposed to RL and BL, compared to WL, for the ‘Pendolino’ genotype. In addition, no substantial variations in the transcript levels of these genes were detected between the two cultivars in plants subjected to WL (Figure 7, Table S1).

As for *PHOT2* and *PHYE*, the expression levels of *CRY2*, *PHYC* and *PHOT1* were also elevated in ‘Canino’ plants exposed to BL and not significantly different respect to the control plants (Figure 7, Table S1). Conversely, for ‘Pendolino’ no significant differences for *CRY2* expression were observed between plants grown under BL and WL (Figure 7, Table S1) or, even, a significant increase in transcript levels of *PHYC* and *PHOT1* was found (Figure 7, Table S1). On the other hand, for all these genes a significant lower expression was observed already in the “usual” conditions (WL) than ‘Canino’ (Figure 7, Table S1).

The other members directly involved in the mechanisms of response to BL were *PHR* and *PHR2* (Figure 7, Table S1). *PHR* expression significantly decreases in ‘Canino’ plants exposed to RL and BL, while remaining constant in ‘Pendolino’ plants under all three experimental conditions (Figure 7, Table S1). Similar behavior was observed for *PHR2* in ‘Canino’ while a significant variation in the levels of this transcript was also observed in ‘Pendolino’. On the other hand, a greater and significant expression was confirmed in plants grown under BL compared to those exposed to RL (Figure 7, Table S1). It is clear that *PHR* and *PHR2* are regulated by BL although they do not exhibit a trend that is strictly analogous to that demonstrated by the other genes belonging to the first cluster described. In fact, although there is a significant reduction in transcript levels in plants exposed to BL, even in cv. Canino, the expression of the two genes remains high (Figure 7, Table S1).

Among the remaining analyzed genes, two of them, *CRY1* and *PHYB*, were more expressed in the cv. Pendolino at all three experimental conditions (Figure 7, Table S1). In ‘Canino’, CRY1 expression was significantly lower in plants exposed to BL than to WL, with no significant differences observed in plants exposed to RL (Figure 7, Table S1). On the other hand, in ‘Pendolino’, no clear alterations in the expression of this gene were observed among the different experimental conditions (Figure 7, Table S1). However, an opposite expression pattern was observed for *PHYB* between the experimental conditions, with significant alterations in transcript levels observed in ‘Pendolino’ rather than ‘Canino’ (Figure 7, Table S1).

Finally, two of the ten analyzed genes for which significantly higher transcript levels were observed in plants exposed to RL than WL, were *PHOT1* and *PHYA* (Figure 7, Table S1). It was observed that the expression of *PHYA* remained constant in ‘Canino’ plants grown under RL respect to WL, while significant reduction in transcript levels was observed under BL (Figure 7, Table S1). On the other hand, this gene was significantly more expressed in ‘Pendolino’ plants exposed to both RL and BL when compared to the respective control plants (Figure 7, Table S1). Furthermore, a lower and significant expression was observed in the “usual” conditions (WL) compared to ‘Canino’ (Figure 7, Table S1).

## 3. Discussion

Light plays a crucial role in plant development and in regulating numerous vital functions, influencing plants both quantitatively, through the duration and intensity of light exposure and qualitatively via different light spectra. While plant responses to various light wavelengths have been extensively studied in numerous plant species, few studies were conducted on tree species [17]. To the best of our knowledge, this is the first research carried out on the olive tree, which elucidated the effect of exclusive and prolonged exposure to RL and BL. Our analysis revealed that exposure to RL alone led to a marked suppression of vital processes in all cultivars, irrespective of genotype. This inhibition ultimately resulted in a complete arrest of vegetative growth by the end of the two-month period. In contrast, plants grown under BL exhibited relatively stable physiological and morphological performance across several cultivars. Notably, however, enhanced responses were observed only in a subset of genotypes, suggesting a cultivar-specific sensitivity to BL.

Based on phenotypic observations derived from the monitoring of various parameters (growth, number of internodes, ETR, GSW, E, and A), it was found that among the ten cultivars analyzed, ‘Arbequina’ and ‘Canino’ exhibited the best adaptation to exclusive exposure to BL. These two cultivars showed no significant differences compared to control conditions two months after the start of the experiment. These findings are consistent with previous studies in other plant species, where key physiological parameters, such as the electron transport rates between photosystem II and photosystem I and stomatal conductance to water vapor, remained stable under BL exposure [22,23,24]. BL is also well-documented as a regulator of plant growth due to its role in photomorphogenesis and phototropism [25]. It promotes biomass accumulation and suppresses hypocotyl and stem elongation [26]. In some cases, it has been observed to enhance cell expansion and division (as seen in lettuce and beans), while in others (e.g., soybean) it has been demonstrated to inhibit both processes [27,28]. Moreover, through involvement of photoreceptors and circadian clock machinery, it affects the production of photo-protective pigments, such as the anthocyanins, and lipid modification, in fruit of many plant species including the olive tree [21].

Therefore, considering our findings, the superior performance of ‘Arbequina’ and ‘Canino’ under exclusive BL exposure, reflected in enhanced vegetative growth and photosynthetic capacity, might be attributed to BL-induced stimulation of cell division. In contrast, under the same conditions, ‘Pendolino’ cultivar exhibited poor vegetative activity, evidenced by low growth, which might be explained by its reduced photosynthetic capacity, as indicated by low electron transport rates, water vapor diffusion, transpiration, and total CO_2_ conductance.

Contrary to the observations made under BL, RL is known to negatively affect similar physiological parameters in cucumber [22,29]. Our observations, which highlight the poor adaptability of all ten cultivars studied to this light spectrum, align with findings in cucumber, yet appear to contradict reports on other plant species where RL has been shown to promote cell expansion and hypocotyl elongation [30,31,32,33]. The poor adaptability of olive to exclusive RL exposure may likely be due to the lack of cell division and proliferation, which cannot be triggered in the complete absence of BL. In contrast, for all cultivars, WL plants showed better vegetative performance, attributable to the presence of both light spectra, which positively regulate cell division and expansion.

An analysis of the expression profiles of ten genes encoding major light receptors in two genotypes, ‘Canino’ and ‘Pendolino’, which exhibited the most contrasting responses to BL exposure, revealed the key role of certain receptors in mediating processes regulated by this specific light spectrum. Some of these findings confirm previous observations in other plant species, while others reveal olive-specific characteristics. Genes *PHOT1*, *PHOT2*, *PHYC*, *PHYE*, *CRY2*, *PHR*, and *PHR2*, which were more highly expressed in ‘Canino’, appear to be primarily responsible for the cultivar’s superior performance under BL exposure. Notably, phototropin 2 is a blue-light-sensitive photoreceptor involved in various physiological processes that collectively support photosynthetic activity under changing environmental conditions; these include phototropism [34], stomatal opening [35], and chloroplast movement [34]. The functions of phytochrome E are also well-documented, including phototropism [36], regulation of internode elongation [37], and seed germination [38]. No significant differences were observed in the expression of this gene in ‘Canino’ plants exposed to BL, like the patterns observed for *PHOT2*. The expression pattern of these two genes correlates with physiological parameter monitoring. The lower expression of both genes in ‘Canino’ and ‘Pendolino’ plants exposed to RL is closely associated with the poor adaptability in terms of vegetative growth and photosynthetic capacity observed in both cultivars. Of particular interest is the significant difference between these two cultivars grown under BL, which appears to be directly related to the marked increase in transcript levels of *PHOT2* and *PHYE* in ‘Canino’. This suggests a potential key role of these genes in regulating important physiological parameters underlying Canino’s superior response to BL. The behavior of *PHOT2* was expected, as it encodes a BL photoreceptor, whereas the observed result for *PHYE*, a photoreceptor typically involved in responses to RL, is rather unusual. Nevertheless, this phytochrome can also be positively induced by BL, influencing phototropic activity and chloroplast movement via phototropins through interactions between photoreceptor families [39,40].

Like *PHOT2* and *PHYE*, the expression levels of *CRY2*, *PHYC*, and *PHOT1* were elevated in ‘Canino’ plants exposed to BL. The advantage expressed by ‘Canino’ over ‘Pendolino’ suggests a key role for these genes in mediating the BL response, which appears to be more strongly amplified in ‘Canino’. This hypothesis aligns with findings in other species, where cryptochrome 2 and phototropin 1 contribute to maintaining physiological responses under BL exposure. Notably, studies in Arabidopsis mutants showed that cryptochromes work additively with phototropins to regulate stomatal opening [41] and chloroplast transcription activation [42]. Both cryptochrome 2 and phototropin 1 are BL-sensitive photoreceptors belonging to different protein families. Cry2 primarily regulates the inhibition of hypocotyl elongation and cotyledon opening [6], controls flowering [43], and, in association with phototropin 1, mediates photomorphogenesis under low blue-light density [43,44]. In tomato, it also contributes to increasing pigment levels such as flavonoids and lycopene in leaves and fruits [14]. Furthermore, phototropin 2 and phototropin 1 triggers numerous physiological responses to optimize photosynthetic activity, including chloroplast migration in response to intense light, stomatal opening, and leaf flattening [45]. Phytochrome C, a RL-sensitive photoreceptor involved in leaf development and expansion [44], also modulates phyB-mediated inhibition of hypocotyl elongation in RL [46]. However, phyC mutants in Arabidopsis have been shown to be hypo-sensitive to BL treatments, suggesting a potential role in modulating hypocotyl elongation even under BL conditions [46].

Considering our findings, it is also evident that genes *PHR* and *PHR2* are regulated by BL, although their expression patterns do not entirely mirror those of other genes mentioned above. Despite a significant reduction in transcript levels in BL-exposed plants, ‘Canino’ maintained relatively high expression of these two genes. It remains unclear whether these are two photoreceptors or photolyases. The conserved PHR motif is shared by cryptochromes, known blue-light photoreceptors, and photolyases, which, while indirectly regulated by BL, do not show functions directly associated with those of light receptors [47].

The opposite trend observed for *CRY1* and *PHYB* suggests an inhibitory effect on vegetative performance mediated by BL. Cryptochrome 1 is involved in development and photomorphogenesis, regulation of de-etiolation, photoperiodic control of flowering, and inhibition of hypocotyl elongation in response to BL [48]. Like cry1, phytochrome B also functions as an inhibitor of hypocotyl elongation in response to both RL and BL [49]. It has been shown that cry1 and phyB work synergistically to control the evolutionary fate of plants exposed to BL, regulating growth and development according to proposed interaction and co-regulation models [10,50]. Our results confirm the synergistic role of these two photoreceptors in mediating BL responses, suggesting a key role in the negative regulation of these processes. Antagonistic actions between phyB and cry2 in Arabidopsis have been observed in photoperiod regulation and floral induction [51,52], as well as antagonistic actions between cry1 and cry2 in root development [53]. Supporting this hypothesis, it has been shown that these two genes are co-regulated by light, maintaining the same circadian rhythm with a peak at the same time of day [20,54]. The synergistic action of cry1/phyB has been studied in Arabidopsis phyB and cry1 mutants, where phyB mutants showed reduced cry1 function, and cry1 mutants exhibited reduced phyB function in hypocotyl response to both red and BL [50]. A cry1/phyB coaction mechanism under BL was also observed in root greening [55] and de-etiolation. Specifically, transcript analysis revealed that expression of certain BL-regulated genes through cry1 persists under RL in the absence of BL, indicating that phyB utilizes the same transcriptional network mediated by cry1, improving light response [10].

Finally, our results confirm the role of phytochrome A in the RL response and in mediating various responses, including growth and development throughout the plant life cycle and de-etiolation under shade conditions [56,57], as previously observed in other species such as Arabidopsis [58], pea [59], and rice [60]. The poor adaptability of olive to exclusive RL exposure may be due to the inability to trigger cell division and proliferation in the complete absence of BL. However, the higher expression of *PHYA* in ‘Pendolino’ plants exposed to RL suggests a key role for this gene in mediating this specific light spectrum and potentially a greater ability of this cultivar to perceive it. Nevertheless, based on the phenotypic data available, we cannot yet confirm this hypothesis, which warrants further investigation.

In conclusion, it is well-established that BL is a growth regulator involved in photomorphogenesis and phototropism, and our observations suggest that this light spectrum could enhance cell division, resulting in improved vegetative growth and photosynthetic capacity. In contrast, the poor adaptability of all ten cultivars to RL is seemingly consistent with the defined role of this spectrum in inducing cell expansion but, in the complete absence of BL, fails to activate and mediate the processes of cell division and proliferation.

The observed differences among RL-, BL-, and WL-exposed plants are primarily attributable to photoreceptor-mediated responses rather than photosynthetic rates, as light functions not only as an energy source but also as a regulatory signal influencing plant development, physiology, and metabolism. These photoreceptor-driven processes could therefore explain variations in plant behavior beyond what can be accounted for by photosynthesis alone. By isolating and characterizing certain genes whose expression patterns appear to correlate with light-mediated quantitative changes, this study provides deeper insight into their molecular bases, paving the way for future work aimed at accelerating and refining olive breeding programs.

## 4. Materials and Methods

### 4.1. Plant Material

Six-months-old olive plants of the ten cultivars Arbequina, Canino, Coratina, Frantoio, FS17, Itrana, Leccino, Maurino, Moraiolo and Pendolino, were grown in pots at temperature of 23 °C and under 16/8 h light/darkness photoperiod with intensity of about 100 µmol m^−2^ s^−1^ provided by Radium Lampenwerk GmbH (Wipperfürth, Germany) 58 W/865 cool daylight lamps (WL). The visible emission spectrum is non-uniform, exhibiting discrete phosphor-derived peaks as reported in the manufacturer’s datasheet: a marked blue/violet band (≈400–450 nm), a dominant green peak (≈500–540 nm), and broader, less intense yellow—orange/red peaks (≈580–620 nm), with negligible emission below 400 nm and above 700 nm. In our setup, four brand-new lamps were installed, providing a PPFD range of ~260–360 μmol·m^−2^·s^−1^, sufficient for six-month-old potted plants positioned ~50 cm from the lamps with a leaf area of ~0.50 m^2^. Plants were acclimated in growth chambers for 30 days. After the acclimation period, the growth chamber was divided into three compartments separated by a screen. Three out of nine plants of each cultivar were placed in the compartment exposed exclusively to red light (RL) provided by Philips (Amsterdam, NL) TL-D Colored 36W Red lamps and three exclusively to blue light (BL) provided by Philips (Amsterdam, NL) TL-D Colored 36W Blue lamps. Four lamps installation resulted in a PPFD range of ~100–224 μmol·m^−2^·s^−1^ for BL and ~160–308 μmol·m^−2^·s^−1^ for RL under these conditions.

### 4.2. Phenotypic Evaluations

After the acclimation period, all plants were marked with colored wire to follow the vegetative growth (T0). One and two months later (T1 and T2) in plants under WL, RL and BL the internode length and neo-formed nodes were recorded. Shapiro-Wilk normality test and Levene’s Test for Homogeneity of Variance from R car package were performed in R (version 4.4.2). Student’s *t*-test was performed by test.t function in Excel by Microsoft Office 365(Microsoft, Redmond, WA, USA).

### 4.3. Physiological Data Evaluation

At T1 and T2, always between 10 am and midday, physiological measurements about transpiration (E), CO_2_ assimilation rate (A), stomatal conductance (GSW) and electron transport rate (ETR) were collected by the LI-6800 Portable Photosynthesis System (LI-COR, Lincoln, NE, USA), in both cases the measurements were conducted on three leaves for each leaf stage: high, medium, low, in three biological replicas for plants grown under each light spectrum. Analysis of variance (ANOVA) using the function *aov*() in R (version 4.4.2) and Tukey HSD test [*TukeyHSD*() from R *car* package] and figures were generated using the ggplot2 package [61].

### 4.4. Quantitative RT-PCR

Three independent RNA extractions from leaves of ‘Canino’ and ‘Pendolino’ at T2, grown under WL, RL and BL with the RNeasy Plant Mini Kit (Qiagen, Hilden, Germany) according to the manufacturer’s protocol, were performed. To remove DNA contaminations, all the samples were processed with the Invitrogen™ TURBO DNA-free™ Kit (Thermo Fisher Scientific, Waltham, MA, USA).

The nucleic acid purity was analyzed via the Thermo Scientific™ NanoDrop™ 2000c Spectrophotometer (Thermo Fisher Scientific, Waltham, MA, USA) and the samples with 260/280 and 260/230 nm absorbance ratios greater than 1.8 were used for the following experiments. Total RNA (100 ng) was used for cDNA synthesis with SuperScript III First-strand synthesis kit (Thermo Fisher Scientific, Waltham, MA, USA) according to the manufacturer’s instructions. The reaction mixes were prepared in three technical replicates in 96-well plates with 5 μL PowerUP SYBR^®^ Green (2X) (Thermo Fisher Scientific, Waltham, MA, USA) and 50 ng of first strand cDNA. PCR conditions were: one cycle at 95 °C for 20 s, followed by 40 cycles of 95 °C for 15 s and 58 °C for 30 s. The melting curve to confirm the presence of a unique amplicon was evaluated and a single peak in every reaction was observed at the end of the PCR. Relative template abundance was quantified using the standard curve method [62] and the Elongation Factor 1-a (EF1A) [63] was used as reference gene for expression normalization. PCR efficiency was estimated using six-point, 10-fold, diluted standard curves. Means from three independent replicates were subjected to SEM calculation and Student’s *t*-test. Ten genes encoding photoreceptors were selected for expression analysis. The primers were designed using the Primer3 software web version 4.1.0 on gene sequences included in a newly reference assembly of ‘Leccino’ (Table S2 and Sequence_S1—https://olgenome.crea.gov.it/index.php?lang=en last access on 11 July 2025).

## 5. Conclusions

This study provided evidences about the effects of the application of different light spectra in olive plants where this kind of approach in such species has been used for the first time. We found that RL and BL caused analogous effects observed in other plants on growth, development and photosynthesis efficiency as well as contrasting effects like in other species. On the other hand, the different cultivars analyzed exhibited distinct responses, suggesting a genetic control of this trait, which, particularly in plants exposed to BL, appears to influence both cell expansion and proliferation. The analysis on photosynthetic activity indicated that an adaptation process of olive plants in response to spectral changes in light occurred as well as in the gene expression of mediators of light perception. The understanding of the behavior of those genes and of cultivars under the two light spectra may represent a key point for future functional genomics studies for genetic improvement of this species.

In fact, the employment of RL and BL can be also considered in the modulation of the plant growth speed, as a strategy for overcoming the long juvenile phase of olive, for more efficient photosynthesis and gas exchange in the nursery context to obtain healthy plants quickly. On the other hand, although this study has provided insights into interesting and novel aspects regarding the effects of different light spectra on a species of significant agronomic and economic importance, such as the olive tree, it represents only the first step in a knowledge-building process that undoubtedly requires further investigation.

## Data Availability

The data supporting the conclusions of this article are available in the Supplementary Data associated with this article and were uploaded to the submission system. Olive cultivars were collected and propagated from the olive germplasm collection field of the CREA-Research Centre for Olive, Fruit and Citrus Crops (Mirto-Crosia, Cosenza, Italy, 3937004.5700 North latitude, 1645042.0000 East longitude). All genetic data are available on EURISCO database (EURISCO Catalogue, http://eurisco.ecpgr.org, date of data consultation (1 September 2025)).

## Supplementary Materials

The following supporting information can be downloaded at: https://www.mdpi.com/article/10.3390/ijms26178319/s1.
