# Correction: Forgione et al. The Effects of the Light Spectral Composition on the Development of Olive Tree Varieties Mediated by Photoreceptors. *Int. J. Mol. Sci.* 2025, *26*, 8319

**DOI:** 10.3390/ijms262311423

**Published:** 2025-11-26

**Authors:** Ivano Forgione, Ida Quattromano, Teresa Maria Rosaria Regina, Amelia Salimonti, Fabrizio Carbone

**Affiliations:** 1 Research Center for Olive, Fruit and Citrus Crops, Council for Agricultural Research and Economics (CREA), Via Settimio Severo 83, 87036 Rende, CS, Italy; 2 Department of Biology, Ecology and Earth Science, University of Calabria—Rende, 87036 Rende, CS, Italy

**Missing Data Availability Statement:**

In the original publication [1], the genetic information for non-model plants and the source of materials was not reported. The correct Data Availability Statement is provided as below. The authors state that the scientific conclusions are unaffected. This correction was approved by the Academic Editor. The original publication has also been updated.

**Data Availability Statement:** The data supporting the conclusions of this article are available in the Supplementary Data associated with this article and were uploaded to the submission system. Olive cultivars were collected and propagated from the olive germplasm collection field of the CREA-Research Centre for Olive, Fruit and Citrus Crops (Mirto-Crosia, Cosenza, Italy, 3937004.5700 North latitude, 1645042.0000 East longitude). All genetic data are available on EURISCO database (EURISCO Catalogue, http://eurisco.ecpgr.org, date of data consultation (1 September 2025)).
