# Supplementary material for: The Effects of the Light Spectral Composition on the Development of Olive Tree Varieties Mediated by Photoreceptors
Source: Int J Mol Sci. 2025 Aug 27;26(17):8319. doi: 10.3390/ijms26178319 (PMC12428501; doi:10.3390/ijms26178319)
Supplement: Supplementary file 1 [file ijms-26-08319-s001.zip › Supplementary_data_rev.docx]

**Supplementary information**

**The effects of the light spectral composition on the development of olive tree varieties mediated by photoreceptors.**

Ivano Forgione^1^, Ida Quattromano^1,2,^ Teresa Maria Rosaria Regina^2^, Amelia Salimonti^1^, Fabrizio Carbone^1,*^

*^1^Research centre for Olive, Fruit and Citrus Crops, Council for Agricultural Research and Economics (CREA), Via Settimio Severo 83, 87036 Rende (CS), Italy*

*^2^Department of Biology, Ecology and Earth Science, University of Calabria - Rende (CS), Italy*

^*^Correspondence: fabrizio.carbone@crea.gov.it;

**Tables**

|  | **Canino** | | | | | | **Pendolino** | | | | | |
| --- | --- | --- | --- | --- | --- | --- | --- | --- | --- | --- | --- | --- |
|  | **WL** | | **RL** | | **BL** | | **WL** | | **RL** | | **BL** | |
|  | **MEAN** | **SEM** | **MEAN** | **SEM** | **MEAN** | **SEM** | **MEAN** | **SEM** | **MEAN** | **SEM** | **MEAN** | **SEM** |
| ***PHOT2*** | 0.1012 | 0.0114 | 0.0479 | 0.0087 | 0.0908 | 0.0261 | 0.1223 | 0.0108 | 0.0250 | 0.0056 | 0.0550 | 0.0097 |
| ***PHY-E*** | 0.0700 | 0.0249 | 0.0305 | 0.0100 | 0.0503 | 0.0116 | 0.0721 | 0.0120 | 0.0356 | 0.0145 | 0.0395 | 0.0057 |
| ***CRY2*** | 0.2095 | 0.0216 | 0.1381 | 0.0325 | 0.1800 | 0.0323 | 0.1080 | 0.0307 | 0.0942 | 0.0083 | 0.0806 | 0.0115 |
| ***PHY-C*** | 1.0078 | 0.1805 | 0.7362 | 0.2456 | 0.7391 | 0.1459 | 0.4492 | 0.0478 | 0.3695 | 0.0541 | 1.0201 | 0.2806 |
| ***PHOT1*** | 0.4100 | 0.0491 | 0.4425 | 0.0797 | 0.4755 | 0.0394 | 0.1697 | 0.0193 | 0.3644 | 0.0495 | 0.4373 | 0.0497 |
| ***PHR*** | 0.2267 | 0.0183 | 0.1321 | 0.0270 | 0.1125 | 0.0218 | 0.0910 | 0.0066 | 0.0799 | 0.0467 | 0.0836 | 0.0049 |
| ***PHR2*** | 0.6072 | 0.0827 | 0.1623 | 0.0206 | 0.3636 | 0.0818 | 0.4960 | 0.0599 | 0.1088 | 0.0075 | 0.2724 | 0.0104 |
| ***CRY1*** | 0.0703 | 0.0120 | 0.0477 | 0.0064 | 0.0446 | 0.0096 | 0.1171 | 0.0257 | 0.0780 | 0.0104 | 0.0805 | 0.0120 |
| ***PHY-B*** | 0.0037 | 0.0015 | 0.0050 | 0.0012 | 0.0032 | 0.0009 | 0.0148 | 0.0031 | 0.0070 | 0.0010 | 0.0108 | 0.0019 |
| ***PHY-A*** | 0.0118 | 0.0041 | 0.0098 | 0.0037 | 0.0040 | 0.0012 | 0.0025 | 0.0009 | 0.0171 | 0.0081 | 0.0100 | 0.0042 |

**Table S1.** Relative expression of the ten genes investigated by RT-qPCR experiments. Means and SEM are reported for ‘Canino’ and ‘Pendolino’ genotypes under WL, RL and BL at T2.

| Code | Annotation | Gene | Primer | Sequence 5' - 3' |
| --- | --- | --- | --- | --- |
| tig02153764g649260 | phytochrome e | PHY-E | Fw | ggccaatgcatgcacaagtaga |
|  |  |  | Rv | ccccttgcagacggatgaattt |
| tig02154217g734170 | phytochrome A | PHY-A | Fw | tggctttgcaggggaaagaa |
|  |  |  | Rv | catccctgcttgcacaagcatt |
| tig00000379g54430 | phytochrome C | PHY-C | Fw | gtggaacgatgcgatgcaaa |
|  |  |  | Rv | tgacccggcagccgaaattat |
| tig00000910_2g109080 | phytochrome B | PHY-B | Fw | agggtggatgagcttagttctg |
|  |  |  | Rv | aatgggagctgttgcagtct |
| tig02154053_2g697650 | cryptochrome 2 | CRY2 | Fw | ttagcgaatgccagtggaacag |
|  |  |  | Rv | gacgagcttgtaggacaaggaa |
| tig00002343g207300 | cryptochrome-1 isoform X1 | CRY1 | Fw | cgatggcttcctgaacttgcta |
|  |  |  | Rv | agggtaattggagccaagctca |
| tig00008969g518620 | (6-4)DNA photolyase | PHR | Fw | taaccatccatccggtcccttt |
|  |  |  | Rv | tcatgctcatcttcgccttcag |
| tig00000366_1g49080 | blue-light photoreceptor PHR2 | PHR2 | Fw | tgcaaccatgggtcagaatgga |
|  |  |  | Rv | tgtgtggttttgccgtgcattc |
| tig00003184g259850 | putative LOV domain-containing protein | PHOT2 | Fw | gcagcgaggcagttgataaa |
|  |  |  | Rv | ccacgaaagaaagggtgctgtt |
| tig00005083g356750 | putative LOV domain-containing protein | PHOT1 | Fw | tgggcccttggcattctgttat |
|  |  |  | Rv | ccttgtgcagaacgttggcaaa |

**Table S2**. List of real-time RT-qPCR primers.

**FIGURES**


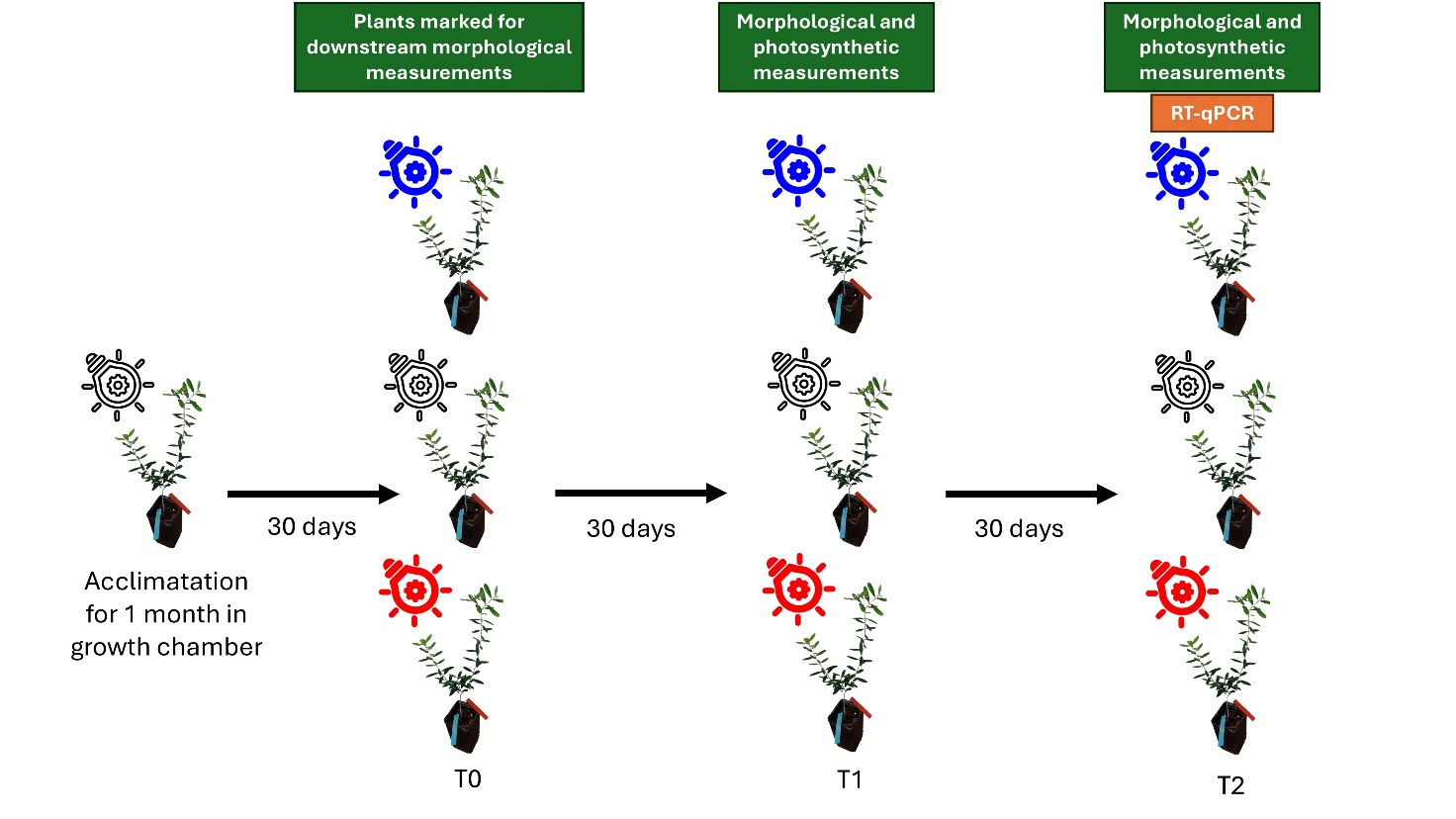


**Figure S1**. Graphical representation of the experimental design. Olive plants were treated with Red light (RL) and blue light (BL) for two months.


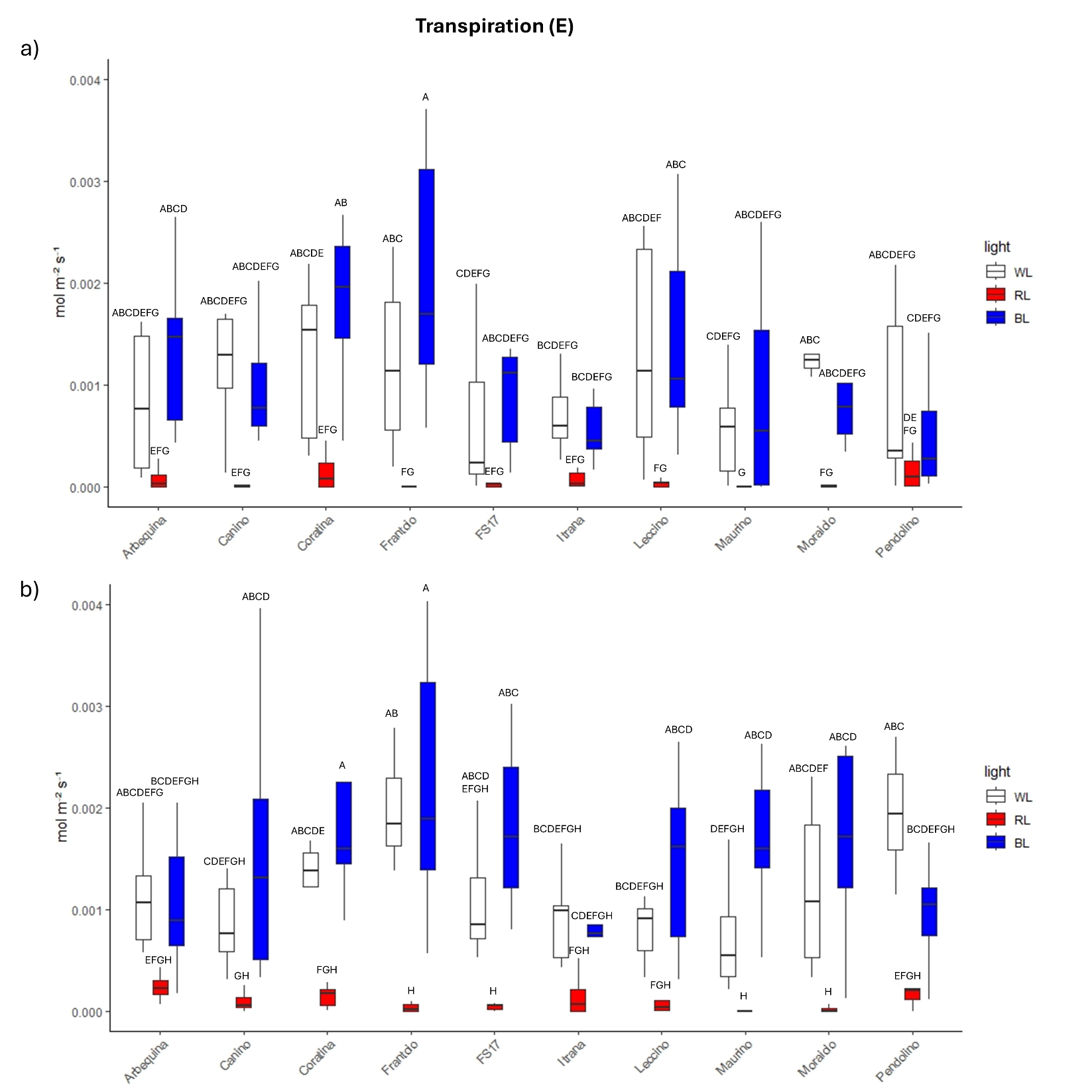


**Figure S2**. Boxplot of transpiration - E in the ten cultivars measured at T1 **(a)** and T2 **(b)**. White, red and blue boxplot indicate WL, RL and BL treatment, respectively. Different letters indicate significant differences within the same time point among all samples according to Tukey’s test (p < 0.05).


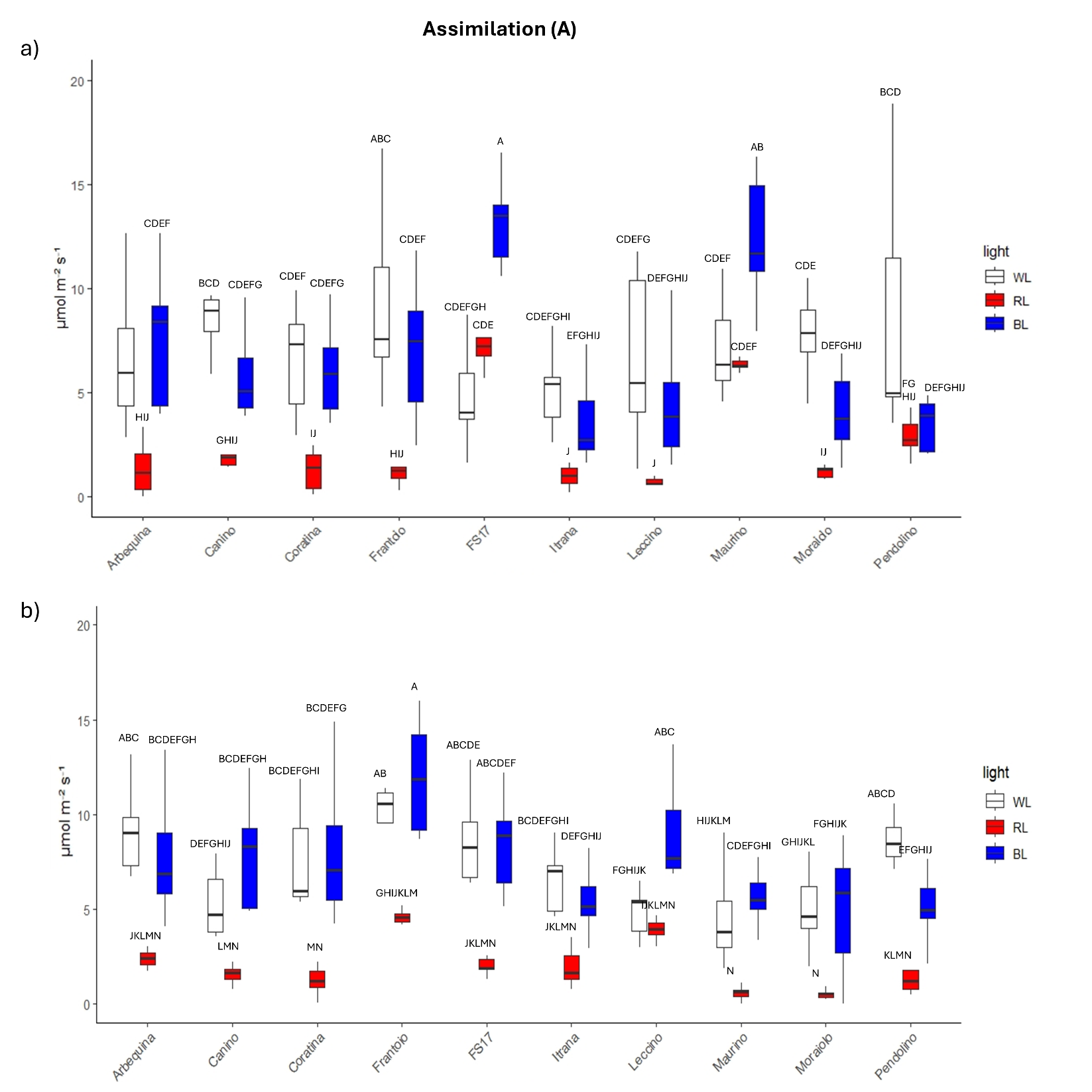


**Figure S3**. Boxplot of CO_2_ assimilation - A in the ten cultivars measured at T1 **(a)** and T2 **(b)**. White, red and blue boxplot indicate WL, RL and BL treatment, respectively. Different letters indicate significant differences within the same time point among all samples according to Tukey’s test (p < 0.05).


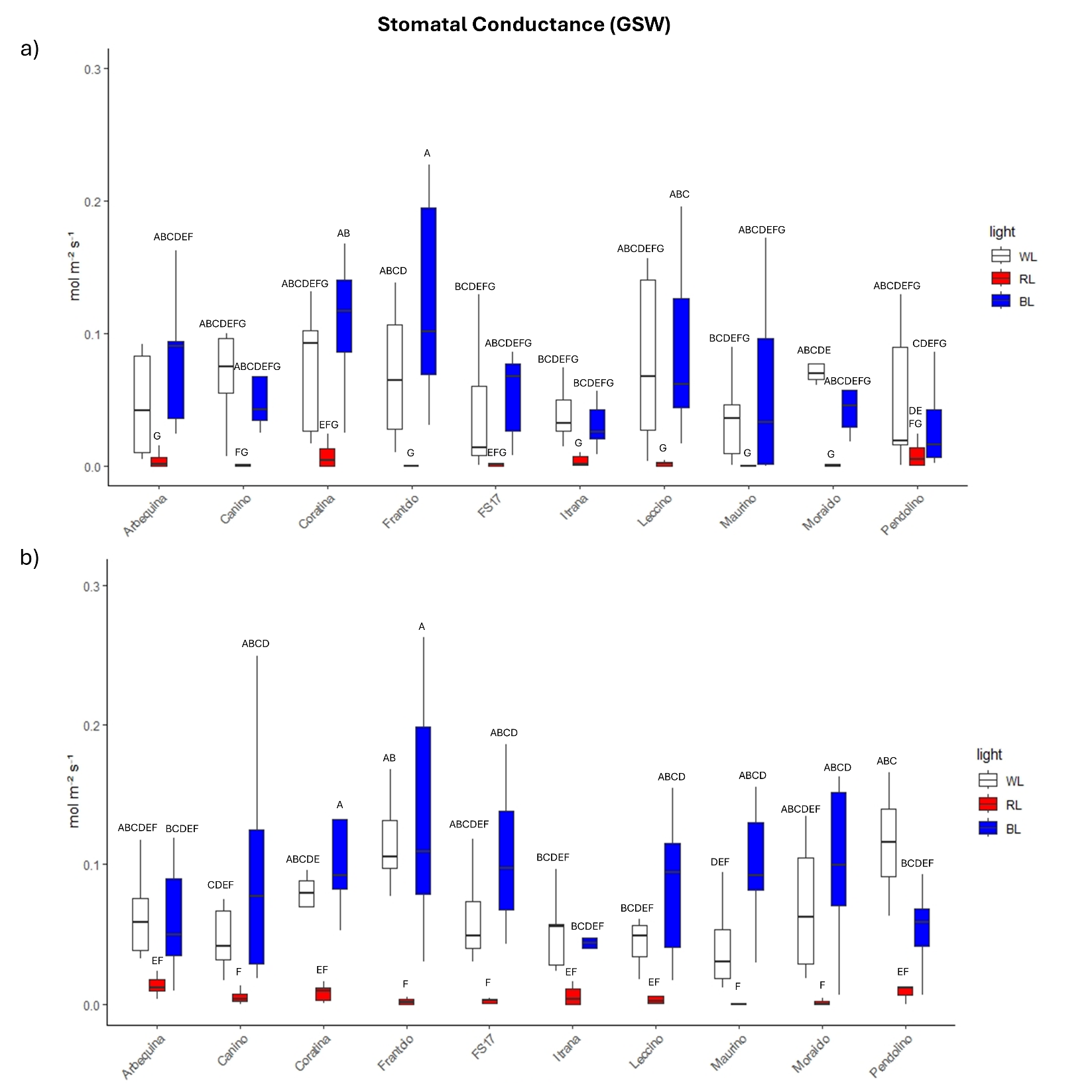


**Figure S4**. Boxplot of stomatal conductance - GSW in the ten cultivars measured at T1 **(a)** and T2 **(b)**. White, red and blue boxplot indicate WL, RL and BL treatment, respectively. Different letters indicate significant differences within the same time point among all samples according to Tukey’s test (p < 0.05).


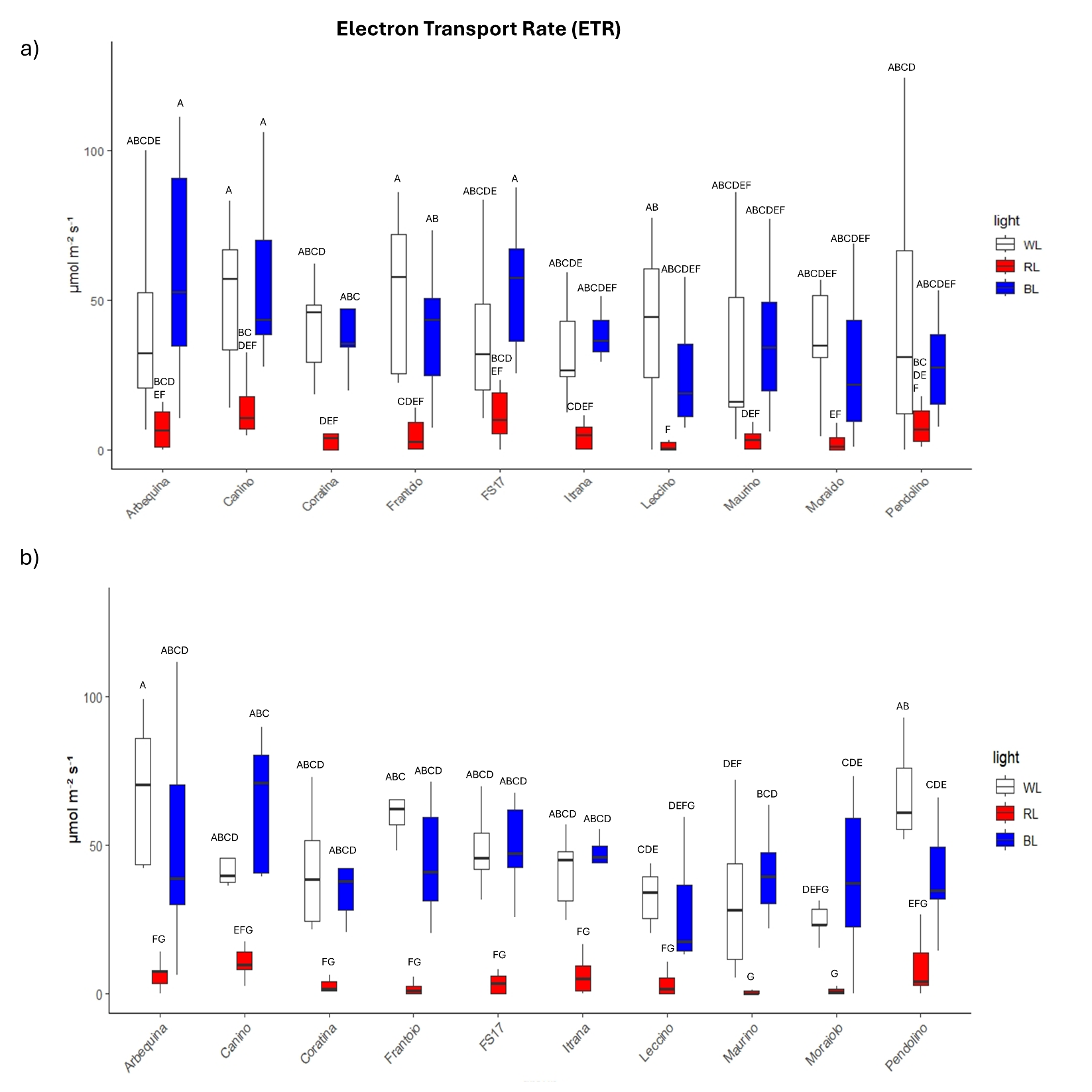


**Figure S5**. Boxplot of electron transport rate - ETR in the ten cultivars measured at T1 **(a)** and T2 **(b)**. White, red and blue boxplot indicate WL, RL and BL treatment, respectively. Different letters indicate significant differences within the same time point among all samples according to Tukey’s test (p < 0.05).
